# Extra Supplement—The Nursing Mirror

**Published:** 1889-04-06

**Authors:** 


### The Nursing Mirror.

All Contributions for this Supplement should be addressed "The Nursing Mirror," care of the Editor, 139-140, Salisbury Court, Fleet Street, London, E.C.

### En Passant.

AN ENTERTAINMENT FOR NURSES.—The nurses of Salford Royal Hospital had a grand entertainment given in their honour last week, which will be long remembered by many of them. The nurses looked very nice in the freshest of uniforms and with the brightest of faces. Many thanks are due to the matron, Miss Penny, and the other ladies and gentlemen who took part in the entertainment.

ADVERTISEMENTS.—We wish to call the attention of our readers to the fact that advertisements are constantly delayed because they are addressed to the Editor, instead of to the Publisher. Before sending an advertisement kindly read the notice at the top of the advertisement sheet of the *Mirror*. We hope our correspondents will also notice that we have moved to new offices at 139 and 140, Salisbury-court, E.C., where all communications should be addressed.

EDINBURGH SCHOOL OF MEDICINE.—Amongst the prize-takers at the Medical School for Women at Edinburgh last month were Alice M. Moorhead, who took the prize for anatomy; Mary H. Cumming, who took the prize for chemistry; Margaret M. Pearse, who took the prize for chemical surgery, and shared the prize for clinical medicine with Jane Marsh; and Jessie M. Macgregor, who took the Arthur Scholarship. The school has just had an anonymous gift of £500, and is altogether in a flourishing condition.

A BRAVE NURSE.—In a book lately published by Mrs. Livermore, in America, called "A Woman's Story of the War," an interesting picture is sketched of "Mother Bickerdyke," a famous character in those times. She was an energetic, sympathetic woman, of slight education, who had a natural aptitude for nursing and an unfailing love of "her boys," as she called all soldiers. Mother Bickerdyke was always to the fore when there was work to be done, and no trials or difficulties ever daunted this brave woman. At first the medical department of the army was grossly mismanaged, and Mother Bickerdyke, with characteristic courage, once persuaded the commander to dress in plain clothes, and then took him to the hospital to see the drunken surgeons. She constantly quarrelled with both doctors and officers, but they all liked and admired her all the same—the men all loved her. After the battle of Chattonooga, she was for weeks the only woman with the 1,800 wounded. The weather was bitterly cold, and the sick were nearly frozen to death in spite of big fires. At last the wood gave out one awful night, and it seemed, indeed, as if those who could not move about would perish of the cold. Mother Bickerdyke had the utmost scorn for red tape, and a mind equal to all emergencies; she called on a few of her faithful "boys" to follow her, and, armed with an axe, proceeded to make firewood of the pallisades. Soon an officer came along and looked on with dismay; there was nothing else would save the wounded, but such irregularity, such rashness, must be punished. "Consider yourself under arrest," he called to Mother Bickerdyke next time she passed him laden with planks. "All right, Major, I'm under arrest; only don't interfere with me till the weather moderates," was the undaunted reply. We wish Mrs. Livermore had given us as brilliant a picture of her own exploits as she has of those of Mother Bickerdyke.

LADY NURSES.—Though the discussion on this subject has ceased in the *Standard*, it has been taken up in one or two other papers. Last week's *The Lady* had an article headed the "Hardships of Lady Nurses," in which much commiseration was wasted on an unexceptionally happy and blessed band of women. By the bye the magazines are particularly devoted to what is certainly the most popular science—medicine—this month. *Harper's* has an article on "The Family Physician," by Dr. Andrew Smith; the *Nineteenth Century* has an article on "Lunatics as Patients, not Prisoners," by Dr. Tuke; and *Blackwood's* contains an article on "The Pleasures of Sickness," by A. Innes Shand.

INSTITUTION NEWS.—The Devon and Exeter Hospital Committee appeal for funds to finish the new Nurses' Home. The governors voted £1,900 last year, but another £700 is needed. The benefit will be not only to the poor, but to the rich of the town, as private nurses will be supplied from the home.—The annual report of the Burton Institution states that 9,384 visits were paid by the nurses last year. There is a balance in hand of £24. Thanks were given to Miss Carson and the staff for the zealous and thorough manner in which they performed their duties. The omnibus company of the town grants free passes to the nurses, which is an example other 'bus companies might follow.—The medical officers of Nottingham Samaritan Hospital state that much of the success from operative work done in the wards is due to the unwearying carefulness of the matron, Miss Nicholas, and to the nurses who so ably assist her.—The Dean of Exeter lately presided at the annual meeting of the Institution for Trained Nurses and Home Hospital, Exeter. There are now twelve nurses in the institution, and they earned £542 last year. When Mrs. Corfe first took the management of the institution it was £600 in debt; but now the debt is paid off, and £300 has been put by to meet future expenses.

THE TEACHING OF NURSES.—One of the most trying duties of sisters and matrons, and one for which they are least prepared, is the teaching of probationers. It is not only that probationers must be shown how "to do things," but they must be coached up for examinations; and as some of them are more or less illiterate the task is not an easy one. Teaching ability is a gift rather to the few than to the many, and is one of the many qualifications a matron should possess. It only exists in a woman who has good knowledge of one subject, thoroughness of instruction, and skill in conducting classes. Governing ability and professional interest are also great helps. The art of questioning is one which is sometimes neglected for the severer but less effectual method of the lecture hall. To fix knowledge in an untrained mind is best done by constant repetition and questioning. Mental activity is also increased by the exercise of answering questions. Illustrations and similes are also very useful in aiding memory; and it is a pity that Latin abbreviations used in prescribing and apothecaries' tables have not been turned into useful rhymes like "Thirty days hath September." It is no use to look on these methods of teaching as babyish; anything which arouses the interest and fixes facts in the memory is not to be disregarded. Every matron or sister whose manifold duties include teaching young nurses should remember that lecturing is but one form of teaching, and should be supported by other and more personal methods. Then her probationers will come out with flying colours at the examinations.

### The Nursing and Management of the Insane.

By T. Duncan Greenlees, M.B. Edin.

*(Being the introductory to a course of lectures to the Nurses of the City of London Lunatic Asylum.)*

Part I.

It is now many years since nurses in general hospitals and infirmaries were first induced to undergo a special training for their duties. In olden times the ordinary hospital nurse was a woman whose brain was continuously in a condition of alcoholic saturation; whose faculties were equally divided between a bottle of gin on the one hand and a state of drunken sleep on the other; and whose real practical knowledge was measured by her own conceit. This nurse, as I have described her, has been faithfully drawn for us by our greatest caricaturist, Charles Dickens, and in Mrs. Gamp we recognise a type of humanity which, let us hope, is now a thing of the past.

Education has effected great reforms in the nursing of the sick, and but for systematic training nursing would still be in the hands of bungling old wives. To Florence Nightingale we are indebted for initiating the noble plan of educating hospital nurses, and the great work, which she commenced amidst the din and clash of warfare, has extended itself until now there is no hospital in this country that does not possess an able and well-educated staff of nurses; and during the past few years numerous excellent manuals on nursing and several periodicals have been published, with the object of extending the knowledge and protecting the interests of those engaged in nursing.

It is only, however, recently that attention has been directed to the education of those having charge of the insane. Many years ago Dr. Browne delivered a course of lectures to his nursing staff at the Crichton Institution; these lectures were afterwards published, but his was an isolated case, and it is to America that the credit is due for the establishment of systematic training schools for nurses who devote themselves to the care of the insane in asylums. America has always been the first to take up any question which has for its elucidation the moral or physical well-being of the race; but in this country, with its old and conservative customs, men are slow to recognise the importance of those subjects which have long been conclusively settled by our transatlantic cousins. Now, however, superintendents of English asylums are beginning to recognise the importance of raising the status of their nursing staffs by lectures and other means, and several years ago the Medico-Psychological Association of Great Britain and Ireland—a society consisting of medical men interested in insanity—deputed a committee of its members to draw up a handbook for the use of those engaged in nursing the insane. This excellent little work, "Handbook for Instruction of Attendants on the Insane" (Baillière, Tindall, and Co.), is well written, and contains sufficient instruction to give its readers a thorough and practical knowledge of their duties.

Attendants on the insane are frequently selected from a class of the community who have had no previous experience either in nursing the sick in a general hospital, or in the management and care of the insane. As a result of this inexperience they are at first entirely ignorant of the various rudiments of nursing, and still less do they know how to deal with patients such as those met with in an asylum. The rules they are expected to read on their appointment are good enough in their way, but are by no means sufficiently explicit to enable them to cope successfully with their numerous and arduous duties.

Life in an asylum to a nurse who takes no interest in her work must be most monotonous, and it is only by educating her mind so that she can appreciate the characters of her patients, and sympathise with their individual idiosyncrasies, that her work will become interesting to her; thereby enabling her to feel that she is fulfilling a noble purpose in caring for those bereft of reason.

Systematic training is the only method whereby we can educate those who have the care of the insane up to that standard of efficiency necessary to a proper and intelligent discharge of their duties. This training should not only be by lectures, but by practical work done in the wards. It is with the purpose of educating your minds to appreciate the importance of your position as nurses, and to make your arduous duties more interesting and consequently easier, that I give these lectures. It is not intended, however, that your training should end in the lecture-room; in the wards, any opportunities that arise for testing your knowledge will be taken advantage of. In this way, if you follow out the advice given here, I will be satisfied, if you can show by "your walk and conversation" that my teaching has not been in vain.

There are still some asylum physicians—no doubt worthy men, but who belong to an old-fashioned school—who maintain that such methods of educating asylum attendants are injurious in the extreme to the interests of an asylum, in so far that they tend to undermine their own authority in the treatment of disease, and in the management of the patients. This, however, is not the case, and it would seem to be merely the expression of fear on the part of timid men; in reality, educated nurses are of great assistance to the physician in the treatment and management of the insane. A knowledge of nursing is a powerful stimulus to an energetic mind, and, in the case of the asylum nurse, will yet raise the status of the office to the level of that of the hospital nurse.

The following remarks on the subject of training generally I quote from the *British Medical Journal:*

"The objects of training are not only to prepare the person to do his work well and with profit to himself, but also to prepare his body and brain for the strains that will be made upon them by the pressure of active life, rendering him apt for the work, full of power and resource, strong, and not easily broken down by temporary trials, adverse circumstances, or overpressure."

*(To be continued.)*

### Notes from Australia.

*(By our own Correspondent.)*

Melbourne, February 8.

I cannot help thinking there is a good opening here for nurses to set up a sort of home for invalids, where every care and comfort would be assured to delicate women, also good nursing, constant care, and massage, or medical rubbing, if ordered. Only it is no use for a nurse to come here without introductions. When I left England everyone offered me introductions to their friends out here, and I only accepted a few, not realising their use. Now I should know better, and accept every such chance which came my way.

The committee of Melbourne Hospital have appointed Miss Haslett assistant matron of that institution, at a salary of £75. Miss Haslett, who has been for twenty years connected with the hospital, is to relieve the matron of some of her work, which is growing beyond the powers of one woman. There is a very big scheme on just now for the enlargement of Melbourne Hospital. It is proposed to get 100 contributors to give £1,000 each, and with this money to erect new buildings to hold from 600 to 800 beds. The secretary has just sailed for Europe, with orders to learn all he can of the latest improvements in hospitals, so that if this scheme is carried out, it may be done on the best and newest principles. Last year 4,216 in-patients and 16,635 out-patients were treated, at a total expenditure of £26,089. Thirty of the doctors who attended the Congress went over the hospital, and were present while Mr. James performed "Emmett's operation" on a patient who has done well.

A private hospital has been lately established at Ballarat, and Miss F. E. Daniels, whom some of your English nurses at Bristol may remember, has been appointed lady superintendent. Ballarat is a very healthy place, situated in charming lake and mountain scenery, and much cooler and more bracing than Melbourne. A little while ago it was impossible to get a trained nurse in Ballarat, and the doctors were thus dreadfully handicapped. Now there are none but trained nurses at this private hospital, and there are telephones to all the doctors' houses. A private hospital of this sort must be much more pleasant than a paying ward of a large general hospital.

The Alfred Hospital has had a large Cinderella ball given in aid of the fund for rebuilding the wing injured by fire. A rough, stormy night, and a bad season for balls—Melbourne folk being mostly at the seaside—made the entertainment less of a success than was anticipated. Tents are to be erected in the extensive grounds of the Alfred Hospital for the reception of typhoid patients. This scheme was tried with success last year, the fresh air circulating amongst the tents having had a most beneficial effect. During the last week of January, 231 cases of typhoid were reported to the Central Board of Health for Victoria, and 24 deaths. This is our periodical outbreak, but it seems more severe than usual. The Alfred Hospital is just now advertising for a secretary at a salary of £350 per annum.

The great sanitary excitement at present is the scheme for selling land adjoining the Yan-Yean Reservoir, which supplies Melbourne and the neighbourhood with water. The whole story is essentially characteristic of colonial cuteness. Many years ago, at a cost of £4,000,000, this reservoir was built; it looks like a beautiful lake, and the ground surrounding it is well wooded and park-like. Not long since a syndicate bought up large tracts of this land at from £30 to £40 an acre, and then offered to sell it to Government at £150 an acre, declaring it was worth that for building purposes. Government felt for a time that it had been done. Of course, dwelling-houses built on the banks of the reservoir would probably pollute the water, and the £4,000,000 be wasted. However, lawyers came forward to state that though houses were built there, the inhabitants would not have the right to row on the lake, to fish, or to drink of its waters; it would merely be a tantalising vision to be gazed at from their windows. There was an attempt to sell the ground in building lots, but a Government official read a proclamation cautioning intending purchasers of the penalties they would incur if they interfered in any way with the reservoir. The sale was a failure. I must mention that two of the syndicate are members of Parliament, and that we pay our members £300 a year to protect public interests!

As an opinion seems to prevail in England that there are no "slums" in Australian cities, I wish to state that Melbourne, at least, can boast that undesirable privilege of poverty. In what is called the "mid-city," where the Chinese congregate, and opium dens abound, there are scenes to be seen quite as sickening and pitiful as any I ever saw in England. Opium and alcohol between them hold undisputed sway over these regions, and at the bars of the public-houses you can see unsexed women, children shapen in iniquity, and palsied old men besotted with drink. Some of the women are great, strong creatures, ripe for any devilry, and absolutely void of all compunction. Here, too, are lodging-houses where a mattress and blanket can be had at fourpence a night; and where the penniless emigrant finds a resting-place amongst old hands who have come down from the bush and spent their pile. Plenty of work here for district nurses, as you can imagine. M. de Freitas, who owns a large restaurant, gives a free dinner every night to all who ask it; he usually gets about 80 guests.

The Medical School of the University is still in an increasingly prosperous condition. The students now number nearly 300, of whom a section are young women. The clinical teaching, as determined upon by the council, appears to be working satisfactorily, both at the Melbourne Hospital and the Alfred Hospital; and the College of Pharmacy supplies pharmacological practice to the satisfaction of the medical faculty. The alleged increasing strictness of the examinations has been occasionally complained of by unsuccessful candidates, but, upon the whole, fair satisfaction is given.

### Nursing in the Soudan.

PART I.—THE START FROM ENGLAND.

SUAKIM and the Soudan are names that have become of late very familiar to English ears, and events still passing there will not permit them to be soon forgotten. I have thought, therefore, that some readers may be interested in the following account of the writer's experiences as hospital nurse during the campaign of 1885.

The story of Gordon's heroic defence of Khartoum stirred the heart of our country with mingled feelings of enthusiastic admiration at his devotion and sadness at his fate. While from time to time graphic accounts reached us of the terrible destruction of Alexandria, the wonderful march of the Camel Corps, the battles of Tel-el-Kebir, Tamai, and Hasheem, accounts of strife, bloodshed, and misery, and scenes amid the dead and dying, so terrible that the imagination cannot add to their horror. There is, however, another part of the story that, as far as the writer of this paper knows, has not been told; and, fortunately for poor human nature, however dark the picture, there is a reverse, with glimpses of brightness; a record of deeds of kindness done by willing hands, of pain and sickness soothed by gentle ministrations, and of sorrows lessened by loving sympathy.

In the early spring of 1885 the Government invited nurses to volunteer for service in Egypt, to supplement, at its expense, the number of those who were already in H. M. Nursing Service. The National Aid Society were also at work securing a band of nurses from various hospitals, to be equipped and despatched at the society's expense, to aid the Government in its humane duty of caring for the sick and wounded. Miss Florence Nightingale's deep interest in all that concerns nursing has in no way relaxed since her memorable work in the Crimea, and none will be surprised to hear that those who took part in the scenes depicted in the following pages were the objects of her tender solicitude in every detail of their work. Help and advice from her were never asked in vain; unending was her kindness and unremitting her efforts for their comfort and well-being, as well as for that of the soldiers who suffered from the cruelties of war.

On March 4, 1885, a party of eight—all volunteers—consisting of four sisters selected by the National Aid Society, and three nurses, with an acting-superintendent, sent out by the Government, set sail from the Albert Docks on board the steamship "Navarino," bound for Suez. The superintendent and three of the sisters to take charge of the nursing department of the Royal Victoria Military Hospital at Suez, the others to receive orders upon their arrival there. The following narrative of experiences has been compiled chiefly from letters written by one of the party.

An account of the voyage out would possess no special interest for general readers; suffice it to say that in due time the party reached Alexandria, and at an early hour in the morning of March 19 we were awakened by the ship moving slowly along in the quiet waters of the harbour of Alexandria, and with a thrill of eager delight gazed through the portholes on the deeply interesting sight, the first view of an Eastern city. Of beauty, Alexandria has not, perhaps, much to boast; but the best view of it is obtained from the sea. The good ship glided along to its place of anchorage by the quay, passed the low-lying lands on either side of the harbour, with the innumerable windmills, the Khedive's palace, and lighthouses. We were soon boarded by officials of all ranks. The very kind and hospitable principal medical officer stationed at Alexandria speedily introduced himself to the sisters of H.M. Nursing Service; offering every facility for seeing the city under his auspices, which, through his kindness, we were able to do. We first paid a visit to Ramleh, going by train over the scene of the campaign of 1882, fortifications and other signs of the late battle being still visible. The Khedive's palace at Ramleh was converted into an English military hospital, the sisters in charge of which were very kind in showing us over and entertaining us.

*(To be continued.)*

### Everybody's Opinion.

*Correspondence on all subjects is invited. No communications can be entertained if the name and address of the correspondent is not given, or unless one side of the paper only be written on.*]

### COMBINATION OF PRIVATE NURSES.

It has been suggested to us that we should act as treasurer for those nurses who desire to combine to secure their own advantage; but before money is sent to us it seems wise to test the general wish by inviting all private nurses who approve the scheme to send their name and address on a postcard, headed "Combination," to the Editor, The Lodge, Porchester-square, London, W. If 200 names are thus received, we shall be glad to arrange for these nurses or their elected representatives to meet together, free of expense to themselves, in order that an accurate idea may be formed of what it is desirable to do in the interests of private nurses as a body. It would be well if nurses would state on the postcards what week day and what hour would be most convenient to them for such a meeting.

### WANTED: SUGGESTIONS FOR A PEACEFUL NIGHT.

N. B. writes: For many months I have been suffering from paralysis of a most painfully sensitive character. I am rendered unable by asthma, and the nature of my paralysis, to lie on either side. A water-bed and air-cushion gives me almost the feeling of living on a bundle of sticks. As night approaches, the whole system becomes intensely saturated with an irritation of an unbearable character, so much so that I have been seriously disposed to urge upon my friends to relieve themselves of their great trouble by placing me in a lunatic asylum. The crisis lasts from about 12 till 5 a.m. I am much too far worn out to devise the scheme that would tend to divert attention from these dreadful attacks. To those who have had the care of such like cases I venture to appeal, having a strong hope that I shall receive some suggestions that will lighten my present misery.

### PRIVATE NURSES.

Contented Ones write: We, the nurses of a private nursing institution in the Eastern Counties, are very sorry to hear that you have so many grumbling letters from nurses, and think it will be a comfort to you to know of some who are satisfied with their home and their wages, as we are, and who love and respect their superiors as we do. We are disgusted to read of a strike amongst nurses being thought of, and would never listen to it. Private nurses, as a rule, have a good home (if they take care to go to a well-known institution), plenty to eat and wear, and soon have a little to spare, which in time accumulates to a nice sum if they are careful, and with that they ought to be thankful, seeing how many deserving struggling people have neither enough to eat nor to wear. Nursing ought not to be looked at in the light of a money-making profession. We ought to think of it seriously, as cherishing God's sick ones.—[Will these nurses still be "Contented Ones" when old age comes, and they have not enough to eat and to wear?—Editor.]

### THE FOOD QUESTION.

Nurse Eva Carter writes: Much has been said in your columns in reference to the "sweating system" with regard to nurses. May I call your attention to the food question? To every true nurse these words will come with a harsh and grating sound. Yet, surely if a nurse is to keep well—if she is to be found equal to any strain or emergency when called upon—she should have, in all fairness to herself and her patients, proper food. By proper food, I refer not to the quantity of it, but to the coarse way in which it is often served in hospitals. I am a private nurse, and in reference to this subject would say that as a rule nurses in private houses have every consideration; not so in many of our hospitals. A few weeks ago I was for four months temporising at a hospital in the absence, through illness, of a charge nurse. The arrangements were as follows: The nurse's breakfast, which usually consisted of bread and very inferior butter, was taken in her bedroom. Dinner at one p.m. This was brought up in a vegetable dish, and consisted frequently of a quantity of coarse, badly-cooked meat, with its accompaniment of vegetable often dabbed on the top, altogether presenting a most uninviting appearance. One has only to picture, what is too often the case, a nurse coming in wearied after a long and tedious operation to find her dinner either dried up or standing quite cold on her table, to prove this statement correct. Nurses do want proper food. Tea and supper (which often consists of bread and cheese only) is taken also in her bedroom. In fact, her bedroom is used both as dining and sitting room. Observation warrants me in saying that my own dinner, and that of other nurses too, often found its way into the pigs' bucket, and the nurses' slender earnings were spent to supplement the loss. Now I maintain that from an economical point of view it would be far better to supply nurses with better food and less of it. And I would add that a dining-room would be much more conducive to the enjoyment of it. At the hospital to which I refer a new matron has arrived, who is doing her utmost to put the wrong right. But I am convinced that the instance I have given is not a solitary exception. I should like to hear the opinion of other nurses.

### Appointments.

[It is requested that successful candidates will send a copy of their applications and testimonials, with date of election, to The Editor, The Lodge, Porchester-square, W.]

St. Cuthbert's Poorhouse, N.B.—Miss Robinson has been appointed first trained lady superintendent of the infirmary in connection with this poorhouse. Miss Robinson will have five nurses under her, and will have to inaugurate scientific nursing. At present Miss Robinson is acting as Sister Aimée at the Edinburgh Royal Sick Children's Hospital.

### Vacancies.

The following vacancies are announced:

### Matrons.

Wrexham Fever Hospital; £30.
Jubilee Hospital, South Kensington

### Nurses.

Holborn Union; £20.

Royal Albert Hospital, Devonport; £25.

Brighton Fever Hospital; £24.

L'pool Infectious Hospital, £25, £35

Eastbourne Nursing Institution.

St. George's Home, Sheffield.

Workhouse Infirmary Nursing Association.

Nottingham Nursing Institution; £25.

Jersey Nurses' Institute; £25.

Nurses' Home, Newcastle; £25.

Liverpool Cancer Hospital.

Canterbury Institute.

Hartlepool Union; £25.

Bridgnorth Union; £25.

Belvedere Hospital, Glasgow.

Nurses' Home, Truro (monthly); £25.

Southport Nurses' Home.

Royal Hospital for the Chest, £22

Kent Nursing Institution.

Kingston Home, Hull.

Sheffield Public Hospital; £22.

Nursing Institution, Ryde.

Nursing Institution, 96, 97, 98a, Wimpole-street, London, W., Mr. Wilson has several vacancies for thoroughly-trained Nurses.

Swansea Nursing Institution; £25.

Miller Hospital, Greenwich; £22.

Barnhill Hospital, Glasgow; £22.

Swansea Hospital; £20.

### District Nurses.

Eardisley; £20.

Madeley, Staffordshire.

West Kent Hospital, Maidstone.

334, Burdett-road, E.

The Grove, Esher.

Hayes, Kent.

### Probationers.

Kent Nursing Institution.
Taunton and Somerset Hospital.
Hospital for Women, Liverpool.
Stoke-on-Trent Nurses Home.

### Notes and Queries.

### Queries.

(1) *Wanted Domestic and Needlework.*—Miss K. Wallis writes: Can any of your readers help me to find a suitable place for a poor woman about 40 years of age, who, owing to bad health, has been out of work for the last two years? She has been a domestic servant, and could now take a light place where housework is required for part of the day, and needlework for the rest. I may mention that she supported herself as a dressmaker for some time. She is thoroughly respectable and trustworthy. Her lungs have been delicate, but she is strong enough for work now, and has no active disease. I ought to mention that she has occasionally had epileptic fits, but for three or four months she has not had one.

(2) *Air Cushion.*—Will anyone send us an air cushion for a chronic invalid at Liverpool?

(3) *Lady Roberts' Nursing Fund.*—All information about Lady Roberts' Nursing Fund can be obtained from Major Cooper, 4, Upper Phillimore Gardens, Kensington, W.

### Answers.

*Nurse L.*—Apply to St. Mary's Hospital, Brighton, or the Broomhill Home, near Glasgow.

*Maureen.*—Pulse 70; respiration 16 to 18. The pulse becomes slower in old people.

### Presentation.

Ayr County Hospital.—On Wednesday, the 27th ult., Miss MacEwen, having resigned her post as matron, a deputation of ladies waited upon her and presented her with a purse of sovereigns in the name of a number of friends. They desired her to accept it as a token of their high esteem and in testimony of their appreciation of her excellent management of this institution.

### THE NURSE'S CASE BOOK.

A Birmingham nurse sends us the following account of a case lately under her charge: About five months ago a doctor in Cumberland was invited to prescribe for a lady suffering from nerve degeneration; six doctors, who had previously had the patient under their care, had retired from the case, stating that nothing could be done for her. The seventh doctor whom the friends of the lady called in, after diagnosing the case, at once decided that massage was the only method of cure from which any benefit might be hoped for. She was then subjected to a course of treatment of complete massage and electricity on the Wier-Mitchell system. The patient had for four years been quite helpless, once only each week lifted out of bed; her voice a low whisper; fits came on about seven each evening, continuing till ten or even eleven; occasionally fits during the night; the patient suffered also from intense headaches; aperients were constantly needed. Looking at it altogether, it seemed quite as hopeless as the six doctors had decided. After fourteen weeks regular daily massage and use of voltaic battery, a result little short of miraculous was produced. The fits were gone, the aperients were unnecessary, the voice returned, and with small assistance the patient could walk about. Weeks have since elapsed. The lady is now quite well.

